# Recent Research Progress of Ionic Liquid Dissolving Silks for Biomedicine and Tissue Engineering Applications

**DOI:** 10.3390/ijms23158706

**Published:** 2022-08-05

**Authors:** Hang Heng, Qianqian Deng, Yipeng Yang, Fang Wang

**Affiliations:** 1 Center of Analysis and Testing, Nanjing Normal University, Nanjing 210023, China; 2 School of Chemistry and Materials Science, Nanjing Normal University, Nanjing 210023, China

**Keywords:** silk fibroin, ionic liquid, dissolution, electrochemical sensor, bone tissue, drug delivery

## Abstract

Ionic liquids (ILs) show a bright application prospect in the field of biomedicine and energy materials due to their unique recyclable, modifiability, structure of cation and anion adjustability, as well as excellent physical and chemical properties. Dissolving silk fibroin (SF), from different species silkworm cocoons, with ILs is considered an effective new way to obtain biomaterials with highly enhanced/tailored properties, which can significantly overcome the shortcomings of traditional preparation methods, such as the cumbersome, time-consuming and the organic toxicity caused by manufacture. In this paper, the basic structure and properties of SF and the preparation methods of traditional regenerated SF solution are first introduced. Then, the dissolving mechanism and main influencing factors of ILs for SF are expounded, and the fabrication methods, material structure and properties of SF blending with natural biological protein, inorganic matter, synthetic polymer, carbon nanotube and graphene oxide in the ILs solution system are introduced. Additionally, our work summarizes the biomedicine and tissue engineering applications of silk-based materials dissolved through various ILs. Finally, according to the deficiency of ILs for dissolving SF at a high melting point and expensive cost, their further study and future development trend are prospected.

## 1. Introduction

The excessive use of non-renewable resources such as oil, coal and natural gas makes us foresee that the scarcity and cost of these resources will bring a huge energy crisis to future society [1]. Biopolymers extracted from natural materials, such as polysaccharides and structural proteins, are the most abundant biomaterials on earth and basic components of life. Among them, cellulose, lignin and xyloglucan are components of vascular plant cell walls [2,3,4], and they are also a kind of dietary fiber beneficial to human health [5]. Chitin is used to provide structural stability and body protection for many insects and can be fabricated to produce scaffolds for the field of tissue engineering [6,7]. Chitosan is a kind of material with high affinity and adsorption capacity for various metal ions, which can be modified into different forms of materials applied in the biomedical field [8,9]. Additionally, keratin is a fibrous desprotein with connective and protective functions, which is widely found in animal hair and toenail [10]. Collagen in the human skin, tendons and ligaments is a major component of the extracellular matrix, which is responsible for maintaining tissue structure [10]. Silk sericin can ensure the cohesion of the cocoon by sticking the twin filaments together [10]. Like keratin from wool or hair, elastin from elastic tissues and resilin proteins from insect tendons [10], fibrin and gelatin have also received much attention in the biomedical and tissue engineering application fields due to their biocompatibility and unique properties as well as high abundance in nature [6,10]. Silk fibroin (SF) exists in arthropods and forms fiber through animal spitting, and the best-known ones are from silkworms [11,12,13,14,15]. SF is a fibrous protein [16,17] containing 18 kinds of amino acids, of which glycine (Gly), alanine (Ala) and serine (Ser) account for more than 80% of its total composition [18]. These highly repetitive amino acid sequences can give protein unique mechanical and architectural properties and promote the protein monomers to self-assemble into structurally interesting hierarchical materials [14]. Hence, SF can provide broad application prospects for the design of new biomaterials due to its biocompatibility, excellent mechanical properties, air and moisture permeability and good processability [11]. After degumming, SF can be prepared into different forms of materials, such as film [19], gel [20], microcapsule [21], 3D printed scaffold [22], fiber [23,24] and micro/nano particle [25] among others. These materials can be applied for drug delivery [21,26], wound healing [20,27], tissue engineering [28,29] and environment-related applications as well as in the energy sector [30]. However, the intrinsic properties of silk proteins depend on their source and processing methods applied [10,11]. Therefore, the dissolution and regeneration of SF have been emerging in several fields.

SF is estimated to have about two-thirds crystalline area with one-third disordered amorphous region. The crystalline region mainly consists of repeated GAGAGS amino acid sequence, connected via hydrogen bond and hydrophobic interaction between molecular chains [31]. Therefore, silk is generally insoluble in most solvents, including water, dilute acid and dilute alkali [32]. Using neutral salt solutions, such as calcium chloride ethanol aqueous solution (CaCl_2_-C_2_H_5_OH-H_2_O) and calcium nitrate methanol aqueous solution (Ca(NO_3_)_2_-CH_3_OH-H_2_O), can bring up a number of problems. For example, the long dissolving time generates an unstable solution that can easily degrade [20,24,33,34]. In addition, these solvents are highly toxic, volatile and difficult to recover, causing serious pollution to the environment. Therefore, it is of great significance to find a new environment-friendly solvent for the regeneration preparation of SF.

**Figure 1:**
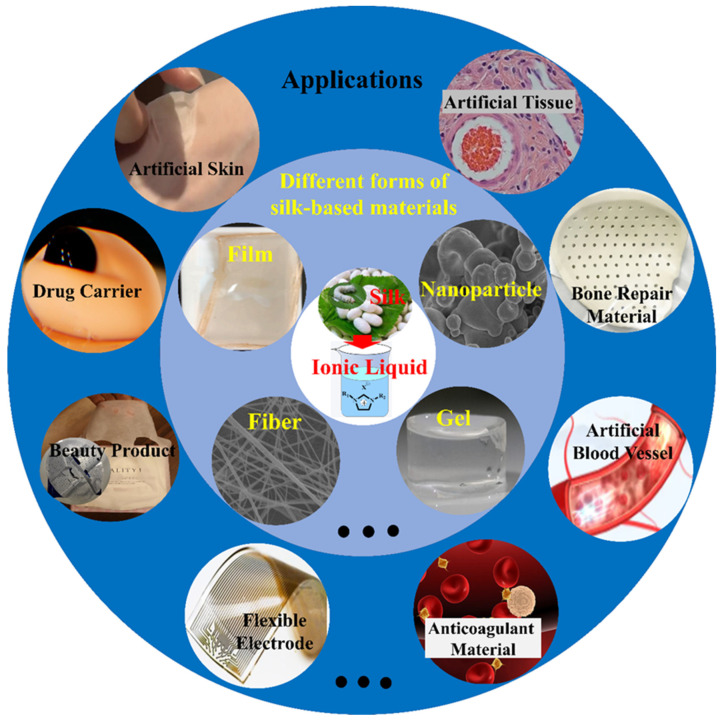
Schematic representation of silk materials and applications after processing with ILs. Silk can be processed into various forms and structures through ILs, such as film, fiber, gel and nanoparticle, which are used as artificial skin, drug carrier, beauty products, flexible electrodes, artificial blood vessels and tissue, as well as anticoagulation and bone repair materials.

Ionic liquids (ILs) are a new type of green solvents developed in recent years, which are molten salts that exist in the form of liquid at or near room temperature. ILs are generally composed of large volumes of organic cations supplying positive charge and organic or inorganic anions providing negative charge [35]. Compared with traditional organic solvents, ILs possess many attractive and unique properties, such as no significant vapor pressure, wide liquid temperature, good conductivity, thermal and chemical stability, and good solubility, as well as designability of structure and characteristics for various materials [36,37,38,39]. The scientific community is increasingly focusing on the advantages, for instance, the high solubility and stability of ILs for silk regeneration, and its composite preparation with other materials is an effective new strategy to obtain various adjustable properties of biomaterials (Figure 1) [40].

Our work mainly reviews several traditional methods of regenerated preparation for SF, systematically expounds on the solubility, dissolution mechanism and characteristics of SF in different ILs, and analyzes the effects of melting point, dissolution temperature and time as well as coagulation bath. At the same time, the preparation methods, material structure, physical and chemical properties and interaction mechanism between SF with natural biopolymers, synthetic polymers and inorganic materials in IL dissolution system in recent years are introduced. The applications of the prepared materials in tissue engineering, drug delivery and electronic engineering materials in recent years are shown. Finally, the shortcomings of ILs in the fabrication of biomaterials are summarized and prospected.

## 2. Preparation Progress of SF Materials

### 2.1. Composition, Structure and Properties of SF

**Figure 2:**
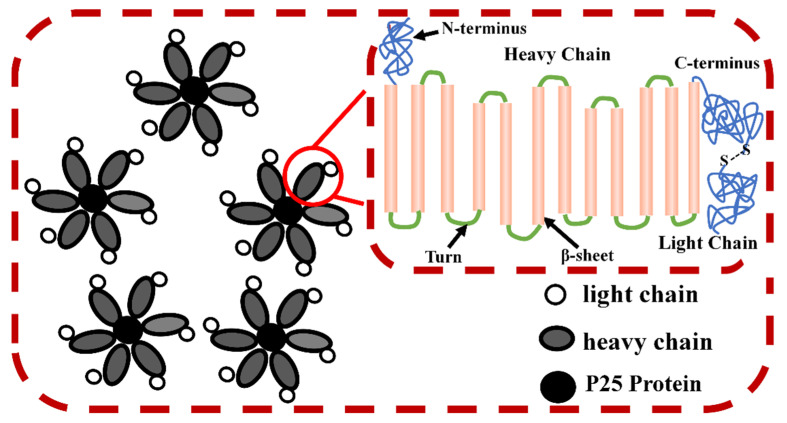
Schematic diagram of the secondary structure of complexation of P25 protein with H chain and L chain in SF. Reproduced with permission from [42], Copyright (2022) Elsevier.

Silk fiber is mainly composed of two core silk protein fibers along with an outer adhesive sericin coat (a set of serine-rich glycoproteins) [28]. Sericin constitutes 20–25% of the weight of the fiber and can be removed by heat or alkaline treatments [28,41]. An SF molecular chain is composed of three components: a heavy chain (H-chain, 350 KDa), a light chain (L-chain, 26 kDa) and a small glycoprotein (P25 protein, 30 KDa) [42]. The light chain polypeptide is linked to the heavy chain polypeptide through a disulfide bond at their C-terminus to form an H–L complex, and is often combined with glycoprotein P25 at a ratio of 6:1 via non-covalent hydrophobic interactions to form a basic micelle unit (Figure 2) [43,44]. In addition, the secondary structure of SF mainly includes *β*-sheet, *α*-helix and random coil structures, among other conformations, in which *β*-sheet is mainly the repeated extension of (GAGAGS)_n_ and (GAGAGY)_n_ in the protein sequence. In the polymerization state, SF is composed of crystalline and amorphous state, and the crystalline state can be divided into silk I and silk II structures [45]. The antiparallel *β*-sheet structure belongs to silk II, which is an orthorhombic crystal system. Silk I is a metastable structure between *α*-helix and *β*-sheet, which is crank-shaped [46]. Silk I may be transformed into silk II structure through the effects of shear force, temperature variations or using solvent [46].

Generally, silk can be found in domesticated silkworms (e.g., *Bombyx mori* silkworm raised in China and in Thailand) and wild silkworms (e.g., Tussah silk produced by the *Antheraea mylitta* silkworm, Muga silk comes from the *Antheraea assamensis* species of silkworm and Eri silk from the *Philosamia ricini* silkworm) [47]. In nature, domesticated silk is produced by artificially bred silkworms and has been used as luxury textiles for centuries. In terms of amino acid composition, domesticated silk is mainly composed of Gly (43–46%), Ala (25–30%), Ser (12%) and tyrosine (Yyr) (5%), as well as valine (Val) (2%). Followed by aspartic acid (Asp), phenylalanine (Phe), glutamic acid (Glu), threonine (Thr), isoleucine (Ile), leucine (Leu), proline (Pro), arginine (Arg), lysine (Lys) and histidine (His), which contents are less than 2% individually [48,49]. The *β*-sheet crystalline and highly ordered crystalline region in SF can be formed through molecular interactions, including hydrogen bond, van der Waals force and hydrophobic interaction between fibroin molecular chains [50,51]. The amorphous region is mainly composed of charged/acidic amino acids, such as Glu, Asp, Arg and Lys [50,51]. Wild silkworm is the general name of *Lepidoptera* silk secreting insects in wild and semi-wild ecology. This kind of cocoon silk has special properties such as moisture absorption, tenacity and strong elasticity. It has also been gradually explored and utilized, and it has become an important biological resource for research on natural silk fiber diversification. 

**Figure 3:**
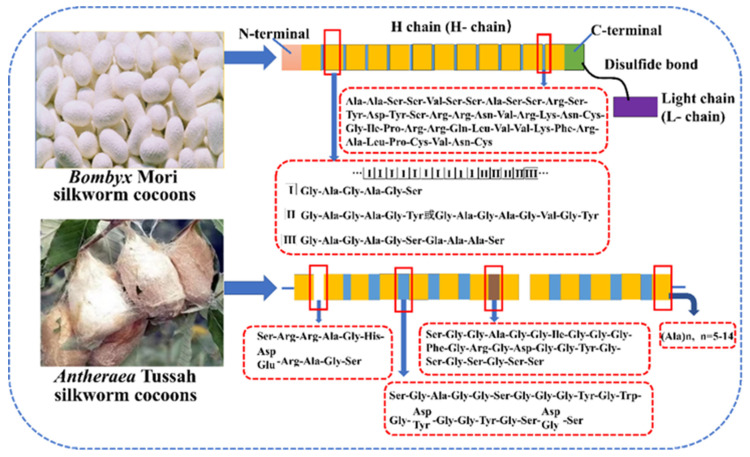
Representative amino acid sequences in SF (H chain) of *Bombyx mori* silkworm and *Antheraea* tussah silkworm (reproduced with permission from [49], Copyright (2016) American Chemical Society and reproduced with permission from [54], Copyright (2022) Elsevier).

Compared with domesticated SF, the wild one lacks an L-chain and P25 protein on the subunit [52]. For example, *Tussah* is one of the most common wild silkworms, which silk primary amino acid composition is significantly different from that of *Mori*. It is mainly composed of Ala (43.07%), Gly (27.27%), Ser (11.26%), Tyr (5.26%) and aspartic acid (ASP) (4.47%) [53]. Its Ala and Asp are richer, and Gly is lower than those of domesticated Mori. [54]. The hydrophobic motif of is mainly (GAGAGS)_x_ for *Bombyx mori* H-chain while polyanaline for *Antheraea pernyi* one [54]. In addition, tussah SF is rich in basic amino acids (Arg and His) and tripeptide sequence Arg-Gly-Asp (RGD), which is a receptor for cellular integrin and is suitable for biomedical applications [54]. The various arrangement order of amino acids determines the difference in SF molecular structure, which can be associated with their mechanical properties and physicochemical properties. Some investigations [54,55] have proved that tussah SF has stronger cell adhesion and excellent mechanical properties than mulberry one, which can be woven into the shape of tendon in tissue reengineering. Figure 3 shows an example of the H-chain amino acid sequence of SF from *Bombyx mori* silkworm and *Antheraea mylitta* Tussah, respectively [56].

### 2.2. Traditional Preparation Methods of SF Regeneration Solution

The option preparation method of SF is to degum the silk, then dissolve and dialysis to obtain the regenerated SF solution, which usually forms an aqueous-soluble protein solution. The selection of solvent is an extremely important parameter in the fabrication of SF-based materials, which will affect its molecular weight [57], the secondary structure, the mechanical properties [50,58] and the physicochemical properties of SF [58,59]. The traditional SF regeneration processing methods include the use of inorganic solution, alcohol mixture solution and inorganic salt system [60]. Several common methods are listed below.

#### 2.2.1. Lithium Bromide (LiBr) Dissolution Method

The LiBr dissolution methods for regenerated SF solution are usually divided into three approaches: (1) 9.0–9.5 m LiBr aqueous solution [59,60,61]; (2) LiBr and ethanol (LiBr-C_2_H_5_OH) mixture [59]; and (3) LiBr-ethanol aqueous solution (LiBr-C_2_H_5_OH-H_2_O) [59,62]. These methods are usually conducted at 40~50 °C and are followed by dialysis in deionized water for approximately 3 days before being concentrated with polyethylene glycol [60,61,62,63]. The concentration of SF solution prepared by these methods is generally in the range of 7~20 wt% [33,64].

#### 2.2.2. Lithium Thiocyanate (LiSCN) Dissolution Method

The degummed SF fiber can be dissolved in LiSCN aqueous solution at a temperate of 40~50 °C. Then, the obtained fibroin solution is dialyzed with deionized water for about 4 days, which normally is aqueous solution [65,66]. The 3~14 wt% SF solution can be generally obtained via this method [65,66,67].

#### 2.2.3. Calcium Chloride Ethanol Aqueous Solution (CaCl_2_-C_2_H_5_OH-H_2_O) Dissolution Method

The degummed SF is dissolved in a CaCl_2_-C_2_H_5_OH-H_2_O solution system for a certain time under heating (40~50 °C) and stirring. As the method mentioned above, the obtained SF solution is dialyzed with deionized water at room temperature for 4 days to remove the salt. After that, the dialyzed solution is stored at 0~4 °C for standby [68]. The SF content can generally get in the range of 3~16 wt% by this way [69,70,71,72].

#### 2.2.4. Calcium Nitrate Methanol Aqueous Solution (Ca(NO_3_)_2_-CH_3_OH-H_2_O) Dissolution Method

The degummed SF is dissolved in a Ca(NO_3_)_2_-CH_3_OH-H_2_O system and heated, then dialyzed with deionized water [59,73]. The method can make the concentration of SF solution reach 2~10 wt% [59].

#### 2.2.5. Calcium Chloride Formic Acid Dissolution Method

The salt acid dissolution system is a gradually developed method for SF fiber in recent years. SF nanofibrils can be obtained by put degummed silk into a calcium chloride formic acid (CaCl_2_-FA) solvent [24]. This solvent can break the hydrogen bond in the crystalline region of silk while retaining its nanofiber structure [24]. The 6~15 wt% concentration for SF solution can be normally obtained in this way [24,74,75,76].

In conclusion, in the process of preparing regenerated SF solution by present traditional methods, it is inevitable to use organic solvents with strong toxicity and volatility, or complex steps such as dialysis and concentration for a long time, and the regenerated SF solution unstable, which SF degradation can easily happen. In order to solve this problem, some researchers [77,78] freeze-dried the SF dialyzed aqueous solution and then re-dissolved it with hexafluoroisopropanol (HFIP) as needed. However, HFIP is also highly toxic and expensive. These facts hinder the large-scale applications of regenerated SF.

### 2.3. IL Preparation Methods for SF Regeneration Solution

People constantly explore and find out that ILs can directly dissolve silk without degradation in SF [79,80,81,82], and the mechanical properties of the prepared SF film can reach more than twice that of the traditional method [83,84]. The electrolytes are typically removed via dialysis against pure water to obtain an aqueous solution of fibroin. The fact that the cocoon can be dissolved directly into ILs is the main reason the processing steps are reduced. In addition, the regenerated SF solution can be quickly separated from the ILs in this solution system with the help of coagulants such as methanol or ethanol, obtain SF materials with various structures and properties, and realize the recovery and reuse of IL, which is a significant advantage of IL [79,80,81,82]. Phillips et al. [79] first found that silk can be dissolved in ILs in 2004. They tested the ability of 1-ethyl-3-methylimidazole chloride ([EMIM]Cl), 1-butyl-3-methylimidazole chloride ([BMIM]Cl) and 1-butyl-2,3-dimethylimidazole chloride ([DMBIM]Cl) to dissolve silk under 100 °C oil bath condition individually and found that the maximum solubility for silk in [EMIM]Cl can up to 23.3%, which is better than that of the traditional methods [79,82,83,84,85]. The second is [BMIM]Cl, which reaches 13.2%, and the last is 8.3% in [DMBIM]Cl. This work shows that ILs have good solubility and dissolution effect on silk. In 2005, they dissolved domesticated silk with [EMIM]Cl and then formed regenerated SF fiber through wet spinning, which can be used as a surgical suture [86]. Subsequently, in 2006, they prepared SF film with [BMIM]Cl, which is conducive to normal cell proliferation and differentiation and can be used as a cell growth scaffold. In recent years, many scholars constantly have been investigating the fabrication methods for dissolving silk with ILs [60,82,87] and trying to apply them to manufacture the SF composites in order to get the required physical and chemical or mechanical properties [49,88,89], which can develop a new way for ILs as a green solvent for natural silk regeneration and blend fabrication.

### 2.4. Mechanism of Dissolving SF via IL and the Influencing Factors

**Figure 4:**
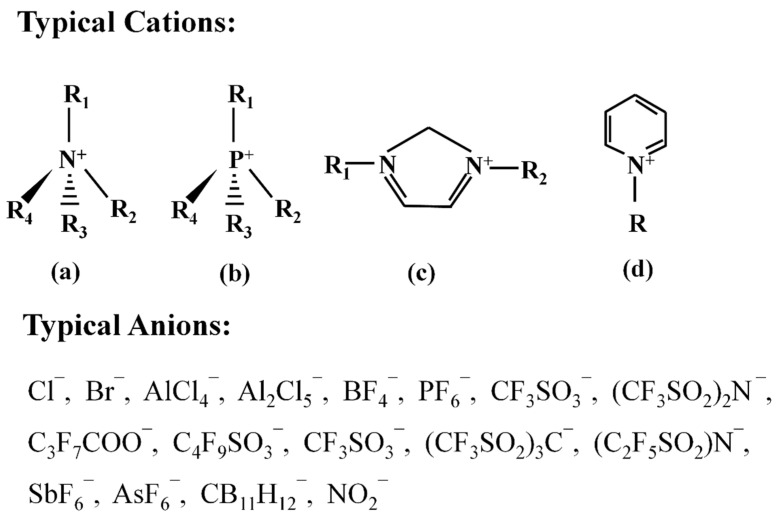
Typical anion and cation structures of common ILs: (**a**) quaternary ammonium salt ions; (**b**) quaternary phosphonium salt ions; (**c**) imidazole salt ions; (**d**) pyrrole salt ions, where R is a common anion.

**Figure 5:**
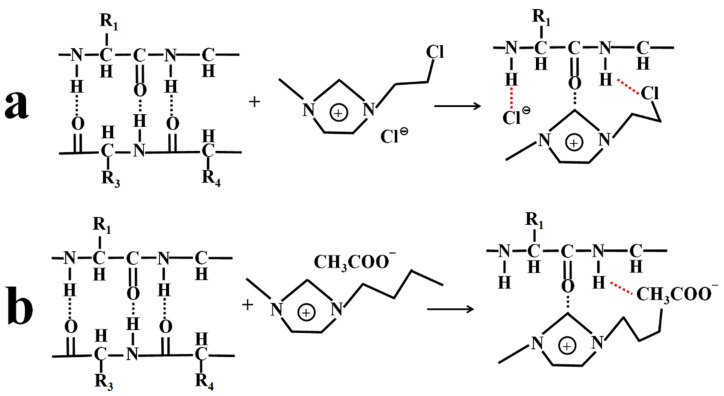
The mechanism of ILs dissolving SF. The −NH of protein chains interact with (**a**) Cl^−^ of [CeMIM]Cl and (**b**) CH_3_COO^−^ of [BMIM]AC to form new hydrogen bonds, which promote the dissolution of SF.

ILs usually contain organic cations and organic/inorganic anions [35]. Typical organic cations include quaternary ammonium salt ions, quaternary phosphonium salt ions, imidazole salt ions and pyrrole salt ions, while the anions include halogen ions, tetrafluoroborate ions and hexafluorophosphate ions, etc. [90] (Figure 4). The properties of anions and cations determine the degree to which ILs can dissolve silk [79,80,81], especially anions [79,80,81]. In these ILs solution systems, anions such as halogen, carboxylic acid and acetic acid in ILs, can interact with N-H in SF, making the initial hydrogen bond between biological macromolecules of SF be broken and silk dissolved. The more anions and cations participate in the action, the greater the degree of destruction of hydrogen bonds between molecules in fibroin and the higher the solubility for SF [79,80,81]. As shown in Figure 5, 1-(2-chloroethyl)-3-methylimidazole ([CeMIM]Cl) contains imidazole cations ([CeMIM]^+^) and chloride ions (Cl^−^). The Cl^−^ in IL can interact with -NH on the SF peptide chains to form a hydrogen bond so as to weaken the original molecular hydrogen bond in SF and finally lead to silk dissolution. Similarly, IL [BMIM]Cl can dissolve SF easily, which is mainly attributed to a strong interaction ability of Cl^-^ from [BMIM]Cl with the hydrogen in the hydroxyl group of SF [79].

Additionally, the binding ability of anions with the SF can affect the solubility of IL to the silk when cations are the same. It has been found [91] that during the process of dissolving SF, the solubility of [BMIM]Cl is higher than that of 1-butyl-3-methyl-imidazole hydrogen sulfate ([BMIM]HSO_4_), which is due to the Cl^-^ possessing a stronger interaction with silk than that of HSO_4_^−^. Xie et al. [83] also found the same phenomenon. They put SF into four kinds of ILs: 1-butyl-3-methylimidazole acetate ([BMIM]AC), 1-butyl-3-methylimidazole chloride ([BMIM]Cl), 1-allyl-3-methylimidazole chloride ([AMIM]Cl) and 1-ethyl-3-methylimidazole chloride ([EMIM]Cl), which solubility was about 10~18% (*w*/*w*). The order of solubility from large to small was: 18.2% for [BMIM]AC > 13.3% for [EMIM]Cl > 12.5% for [AMIM]Cl > 10.4% for [BMIM]Cl. The anion CH_3_COO^−^ in [BMIM]AC is considered to be a coordination base with a low molecular weight, which interacts easily with the hydroxyl group in SF [79]. In addition, the physical properties of ILs, such as melting point (mp.), also affect the solubility of SF [81]. According to the scientific reports [81,92], both [EMIM]Cl (mp. 89 °C) and [BMIM]Cl (mp. 65 °C) dissolving SF need at a high temperature, at about 100 °C, while [AMIM]Cl can dissolve SF fast at ambient temperature, owe to its low melting point, only 17 °C, which can prevent SF molecular chains from degradation at a high temperature and decrease the mechanical properties of regenerated fibers. Therefore, IL with a low melting point is undoubtedly an excellent solvent for SF. 

Therefore, the solubility of SF in ILs depends not only on the action of anions and cations, as well as their physical properties, but also on the temperature and time. Ren et al. [93] dissolved SF with [BMIM]AC at the temperature from 70 °C to 110 °C, respectively. They found that the dissolution rate increased from 5 mg/min to 30 mg/min, and the time required for complete dissolution decreased from 60 min to 10 min with the temperature increasing. Excessive temperature will cause SF degradation. Therefore, 75 °C was selected as the best dissolution temperature for SF. At this temperature, the solubility increased from 1% to 15%, with the increase in dissolution time from 30 min to 840 min. Zhang et al. [94] also demonstrated that the temperature has an important effect on the dissolution of SF in [BMIM]Cl. They showed that the SF was difficult to dissolve in [BMIM]Cl even if the dissolution time was prolonged when the temperature was less than 90 °C. While above 90 °C, silk was dissolved easily, and its dissolution rate can increase significantly with the increase in temperature. However, the fibroin degradation can be raised with the dissolution temperature increasing and the time expansion. Hence, it is of great significance for the selection of optimal conditions of dissolution to maintain the integrity of the heavy and light chains in SF [95].

**Figure 6:**
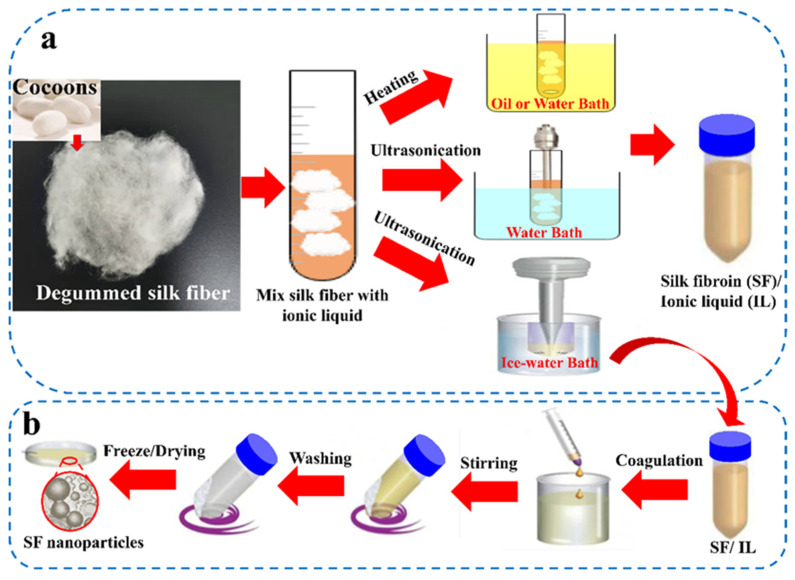
(**a**) Several different heating methods for dissolving SF with ILs: water or oil bath under vortex stirring, dissolving in water bath and in ice water bath by ultrasonic treatment, respectively. (**b**) The whole process of preparing SF nanoparticles by dissolving SF after ultrasound in ice water bath. (Reproduced with permission from [82], Copyright (2022) Elsevier, and reproduced with permission from [95], Copyright (2020) American Chemical Society).

Since SF is a highly crystalline hydrophobic protein with extensive hydrogen bonds [93,94,95], some researchers would also use helper vortex stirring or ultrasound in order to accelerate the solubility of SF in ILs, which was obtained the stability and excellent mechanical properties of the silk protein [82,96]. As shown in Figure 6a, degummed silk is mixed with IL and then put into a water bath or oil bath for vortex stirring or ultrasonic treatment to achieve rapid and efficient dissolution for SF [82,96]. High power ultrasound has two important synergistic effects on the mixed ILs system: the ultrasound can rapidly increase the temperature of ILs and helps to introduce the ILs into the cohesive SF network and disrupt its hydrogen bond until SF completely dissolution [97]. The research [80] also shows that the effect of ILs on hydrogen bonds in *β*-sheet conformations can be enhanced under high-power ultrasound and significantly shorten the dissolution time. In the study by Carissimi et al. [87], in order to realize the dissolution for SF through IL 1-ethyl-3-methylimidazole acetate ([EMIM]AC) at relatively low temperature (below 75 °C), avoiding protein degradation, ultrasound help treatment was carried out during the dissolving process. The SF solubility reached 10% (*w*/*w*). Fuster et al. [85] also used a similar method to rapidly dissolve SF in [EMIM]AC. After rapid desolvation with methanol and being adsorbed with naringin (5,7-dihydroxy-2-(4-hydroxyphenyl), which possessed a wide pharmacological effect, SF nanoparticles loaded with naringin drugs were successfully obtained. This material has higher in vitro antitumor ability than free naringin for HeLa cancer cells [34]. Additionally, as shown in Figure 6b, SF and IL mixed solution prepared by ultrasound was quickly injected into excessive polar organic solvents such as acetone, ethanol or methanol to form SF suspension, to be easily separated from IL, and then SF nanoparticles could be obtained via centrifugation or filtration [97]. In this process, the protein chains would reconstruct the hydrogen bond network and change from random coils structure to a highly ordered *β*-sheet conformation structure. Compared with the traditional methods above, this preparation method significantly reduces the time and steps required to form SF nanoparticles [87].

Just as the above said, the SF solution dissolved in ILs can also easily form films through coagulants such as methanol, acetonitrile and ethanol [98], which can induce the secondary structure of SF transform and achieve the strengthening of mechanical properties of materials [88]. Phillips et al. [79] dissolved SF to form a 9.51% (*w*/*w*) solution by using [BMIM]Cl and followed by casting to a film, the acetonitrile, methanol and water as coagulation bath were used. It was found that the more *β*-sheet structure conformation of SF was induced by methanol than that by acetonitrile. Compared with domesticated silk, tussah silk has higher biological activity, but there are few reports on it. Goujon et al. [99] dissolved the semi-wild *Antheraea assamensis* silk by using IL [BMIM]AC to form SF films. Their research illustrated the different coagulant effects on the *β*-sheets content in the SF film, the order was: isopropanol ethanol (50:50) > isopropanol > water > isopropanol methanol (50:50) > ethanol > methanol. They pointed out furtherly that the *β*-sheet content in SF was affected by the morphology, thermal and mechanical properties, as well as the conductivity of materials [88].

At present, biological functional materials [86], energy materials [100] and environmental protection materials [96] with various properties were attempted to be fabricated constantly through ILs usage. For example, green SF-based films with good mechanical properties, high conductivity and electrochemical stability have been produced by the use of IL 1-butyl-3-methylimidazole hexafluorophosphate ([BMIM]PF_6_) as solvent [100]. The SF/1-ethyl-3-methylimidazolium acetate ([EMIM]AC/H_2_O/KCl) hydrogel could be prepared by taking advantage of the notable feature of IL, such as non-volatile, low freezing point and good conductivity [86]. This produced system processes excellent mechanical properties, water retention, frost resistance and conductivity and can be used in artificial skin, drug release materials and bone tissue engineering, as well as the development of new applications in harsh environments [96,101]. Moreover, ILs can also be used to disperse the SF components well and stabilize the nanoparticles in an aqueous solution, which can be applied in the surface modification of anti-ultraviolet skin care products and other industrial materials, as well as drug or enzyme carrier fields [87,97]. Simultaneously, people are also exploring to manufacture the various silk-based composite materials with various desirable physical and chemical properties using the ILs dissolution system. In the following, we will discuss a variety of topics related to the use of ILs as the main solvent to dissolve silk-based composites and their applications for biomedicine and tissue Engineering, including silk-based blends and composite materials with natural biopolymers, synthetic polymers and inorganic materials, as well as the material structure, the properties and interaction mechanism. Table 1 lists a summary of different applications in biomedicine and tissue engineers of SF-based composites through using various ILs in recent years. 

## 3. SF-Based Composites Prepared Using ILs for Biomedicine and Tissue Engineering Applications

SF-based materials can be fabricated into various forms with specific properties applied for biomedicine and tissue engineering by using ILs solution, such as the thin film with hydrophilic properties for artificial skin coating [97], the gels with conductive and rheological properties for biomedical sensors [49], the scaffolds with high mechanical and cytocompatible properties for bone tissue [82] and the nanoparticles with stability properties for drug carriers [85]. 

### 3.1. SF/Natural Biopolymer Blends and Composite Materials

Cellulose is one of the most abundant natural polysaccharides in nature, which is considered an almost inexhaustible raw material and widely exists in trees, plants and fruits [118]. The molecular structure of cellulose consists of repeated β-d-glucopyranose via covalent action through acetal function, which contains a large number of hydroxyl groups. This type of molecular structure gives cellulose unique properties, such as hydrophilicity, chirality, degradability and easy chemical modification [119]. Some researchers [103] explored cellulose and SF composites using used ILs to obtain the unique physical and chemical properties and biocompatibility. Shang et al. [105] fabricated SF/cellulose composite film with hydrophilic [BMIM]Cl and found that the interaction between SF and cellulose in the blending process would induce its conformation change from silk I structure to silk II. When the mixture ratio of SF to cellulose was 25:75, the tensile strength of this film reached 11.5 ± 1.1 MPa, which was the highest in all film samples. Meanwhile, its aqueous stability is also high, and it can be insoluble in water for 240 min. Tian et al. [106] studied the conformation and intermolecular interaction between cellulose and SF blend membrane prepared with [BMIM]Cl by using high-resolution solid-state NMR. They believed that the strength and toughness of the blend membrane are better than those of the regenerated cellulose membrane, which was attributed to the hydrogen bond action between the -NH group in SF and –OH in cellulose during the blending process. Compared with the pure SF membrane, the content of *β*-sheet structure in the blending contained more, which could be used in biochemistry and biomedicine applications. 

**Figure 7:**
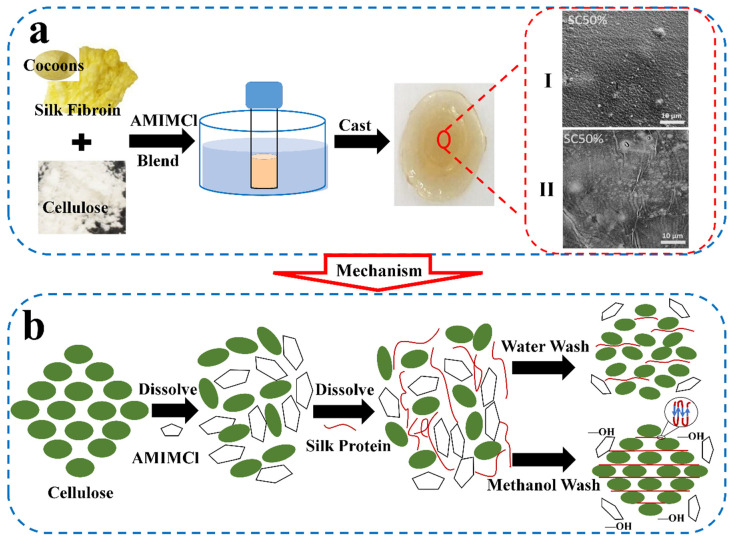
(**a**) Schematic diagram of preparing process of SF/cellulose film using IL [AMIM]Cl as solvent. The SEM images of blends with different mass ratios under methanol treatment (I) and water treatment (II) are shown. (**b**) The blending mechanism in IL [AMIM]Cl system (reproduced with permission from [103], Copyright (2017) Elsevier).

The basic steps of preparing SF/cellulose composite film with IL as solvent are shown in Figure 7. Firstly, silk and cellulose with different mixing ratios were dissolved in [AMIM]Cl, then solidified and washed to form a blending film. The surface morphology of the films treated in the methanol bath (Figure 7a(I)) and water bath (Figure 7a(II)) individually exhibited different surface morphology. Furthermore, the cation and anion in IL combine with oxygen and hydrogen in cellulose hydroxyl, respectively, interrupting the hydrogen bond and leading to cellulose dissolution (Figure 7b). Then, the cellulose structure becomes disordered, the fibers swell and interacts with SF molecules. After washing and solidification, cellulose will return I crystal structure and insert into SF molecules. At the same time, the SF component will self-assemble to form a large number of *β*-sheet structures [88,104,120,121]. Compared with a water bath, the films prepared in methanol bath have higher strength and hardness, mainly due to more *β*-sheet crystals in SF by methanol inducing. In addition, various physical properties of the material can be regulated by changing the blending ratio between silk and cellulose. For example, the mixed film with cellulose content > 50% has better hardness, flexibility, better thermal stability and water insolubility, while the mixed film with SF content ≥ 70% has better elasticity and water solubility. Moreover, through theoretical modeling, molecular dynamics simulation and experimental technology using, Hadadi et al. [90] studied the regeneration mechanism of cellulose/SF, chitin/SF and chitosan/SF biological composites dissolved in ILs. They demonstrated that the strong interaction between the anions in the ILs and the hydrogen bond receiving functional groups on the biopolymer molecular chains could weaken the networks of intermolecular and intramolecular hydrogen bonds in SF or polysaccharide. When water was used as the coagulant, the anions migrated from the biopolymer, blending into the water, and the aggregation of the polymer chains could be started. Simultaneously, this competition between water molecules and different biopolymers to form hydrogen bonds could result in phase separation and the formation of hydrogels (biopolymers + water) and liquid phases (water + ILs). This “gel” process was not caused by chemical crosslinking but by competition between different interactions. Finally, the intercalation structure could be formed. 

As mentioned above, it is the key to controlling the physical and chemical properties of protein and polysaccharide biological composites to select appropriate ILs and coagulation baths, such as the solubility, glass transition temperature, conductivity, thermal stability and mechanical property [88,89,90]. Stanton et al. [89] fabricated SF and cellulose blends using a class of ILs with similar structures and different alkyl chains, such as 1-allyl-3-methylimidazole chloride ([AMIM]Cl), 1-ethyl-3-methylimidazole chloride ([EMIM]Cl) and 1-butyl-3-methylimidazole chloride ([BMIM]Cl), as well as another type of ILs containing larger anion groups, such as 1-ethyl-3-methylimidazole acetate ([EMIM]AC), 1-butyl-3-methyl-imidazolium bromide ([BMIM]Br and 1-butyl-3-methyl imidazolium mesulfonate ([BMIM]MeSO_3_). The mixed films prepared with [AMIM]Cl, [EMIM]Cl and [BMIM]Cl as well as [EMIM]AC were firm and transparent, while the films prepared with [BMIM]Br and [BMIM]MeSO_3_ are translucent and fragile. The film topology prepared by IL with chloride ion group looked more uniform, while the one prepared by IL with bromine ion or mesylate group looked like a fiber structure. The film materials prepared with larger anions have more *β*-sheet, while the crystallinity of the film containing chloride ions is the lowest. The order of *β*-sheets content was [BMIM]MeSO_3_ (58.9%) > [BMIM]Br (58.6%) > [EMIM]AC (39.2%) > [BMIM]Cl (37.5%) > [EMIM]Cl (37.1%) > [AMIM]Cl (31.0%). These changes are related to the number of hydrogen bond disruptions in the anti-parallel *β*-sheet structures, to which extent was affected by the interaction between ILs and natural polymers. Additionally, the initial decomposition temperature of [EMIM]Cl film is about 40 °C higher than that of [EMIM]AC film, and the stability of films prepared by [BMIM]Br and [BMIM]MeSO_3_ with larger anions was higher than that of chloride ion films because larger anions have more interaction sites to make the thermal stability of the film better. Therefore, the thermal stability of materials depends more on the type of anions. 

Some researchers [88] selected [EMIM]AC and [EMIM]Cl to dissolve SF/cellulose (50/50 *w*/*w*) blend individually, and 25% ethanol and 25% hydrogen peroxide (H_2_O_2_) as coagulation bath, respectively. They found that different morphology for all samples appeared. For example, the [EMIM]AC film regenerated with ethanol ([EMIM]AC-Et) has a ridge running through the whole film surface, while the [EMIM]Cl film regenerated with ethanol ([EMIM]Cl-Et) is relatively smooth. The [EMIM]AC film regenerated with H_2_O_2_ ([EMIM]AC-H_2_O_2_) has a shallow sphere with a long ridge, while the [EMIM]Cl with H_2_O_2_ ([EMIM]Cl-H_2_O_2_) was much smoother. For the *β*-sheet content of four film samples, the trend was found in the order of [EMIM]Cl-H_2_O_2_ film (48.5%) > [EMIM]Cl-Et film (46.2%) > [EMIM]AC-Et film (41.0%) > [EMIM]AC-H_2_O_2_ film (14.3%). In terms of thermal properties, the [EMIM]Cl-Et sample has the highest initial decomposition temperature, the lowest mass loss rate and the highest glass transition temperature in all samples, while the elastic modulus and hardness of [EMIM]AC-H_2_O_2_ film were significantly increased. They believed that the crystallinity of film depended on the used coagulant, while it has nothing to do with which IL was used. Another study by them [107] has found that 10% ethanol could induce more *β*-sheet content than that of 1% ethanol used, which could also affect the conductivity of the biological composite film. Dielectric relaxation spectra results showed that the higher the *β*-sheet content was, the higher the conductivity of the sample was [107,122]. The research from Love et al. [108] indicated that the change of H_2_O_2_ coagulant concentration, such as from 1 to 25% (*v*/*v*), has no effect on the *β*-sheet structure for SF in SF/cellulose. However, with the increase of H_2_O_2_ concentration, the cellulose in the blend could gradually change from amorphous to semi-crystalline structure, and its grain size also became large, and the film solidified with hydrogen peroxide has better thermal stability than the one solidified with water. 

Most of these researches mainly focus on film and gel, as well as the blending of SF with cellulose in ILs, and preliminary exploration of the solubility and various properties of material in IL solution. However, recent research [110] reported that the scaffolds from sucrose acetate isobutyrate and SF, through dissolving in [BMIM][Ac], might be offered as an alternative for sick tissue. A bioactive sponge fabricated with silk and bio-IL (gallate and cholinium hydroxide (Ch[Gallate])) could be used in accurately targeting pathologies treatment [111]. Shamsuri et al. [109] expounded that cellulose and silk could be blended into fiber membrane by dry-jet wet-spinning technology using [BMIM]Cl as a cosolvent. The chitosan/silk blend membranes and hydrogels could be fabricated through [AMIM]Cl and [BMIM]Ac, respectively. These biomaterials can be used as biomedicine and tissue materials. 

In addition, the research [99] reported that conventional solvents for Bombyx mori silk could not dissolve *Antheraea assamensis* SF, while [BMIM]Cl could dissolve natural fibers for 12.2 wt%, [BMIM]Ac for 10.14 wt%. Zhang et al. [49] found that using [EMIM]Ac solution, the gelation of *Antheraea pernyi* silk was easier than that of Bombyx mori one because of the very high hydrophobicity of *Antheraea pernyi* contributed by the polyalanine sequence. This kind of material can be used as biomedical hydrogels. DeFrates et al. [103] used [AMIM]Cl to blend Thailand gold Bombyx mori silk and cellulose blend films, which can be applied as a biomaterial with good biocompatibility and degradability in the field of biomedicine. Silva et al. [102] developed [BMIM]Ac IL to fabricate Eri silk (non-mulberry silkworm. ricini, white variety) silk gel and sponge as a candidate for cartilage-related biomedical application. The solubility is up to 10%, similar to the Bombyx mori one.

### 3.2. SF/Synthetic Polymer Composite Materials

How to manufacture polymer biomaterials with excellent mechanical properties has always been a challenging topic [123]. Relevant studies showed that the mechanical properties of SF were significantly improved after compounding with synthetic polymers such as polylactic acid, polyvinyl alcohol and polyurethane [112,113,114,124]. It has been reported that [113] by dissolving degummed silk and polyurethane (PU) in 1-butyl-3-methylimidazole chloride ([BMIM]Cl) and N-N dimethylformamide (DMF) system, a water-insoluble biological composite membrane with good hydrophilicity and biocompatibility could be obtained. Both SF and polyvinyl alcohol (PVA) can also be dissolved in 1-allyl-3-methylimidazole chloride ([AMIM]Cl) to form a biological blend film, avoiding the use of toxic agents such as formaldehyde and glutaraldehyde [114]. Our research group also conducted a similar study [112], using 1-butyl-3-methylimidazole chloride [BMIM]Cl and N-N dimethylformamide (DMF) to dissolve degummed silk and poly(d,l-lactic acid) (PDLLA). The addition of SF significantly improved the hydrophilicity and biocompatibility of PDLLA, increased *β*-sheet content and promoted the self-assembly of micelle structure. This study demonstrated that the morphology, structure, and physical and biological properties of the composite can be regulated by changing the blending mass ratio of SF and polylactic acid in the IL system.

### 3.3. SF/Inorganic Composite Materials

**Figure 8:**
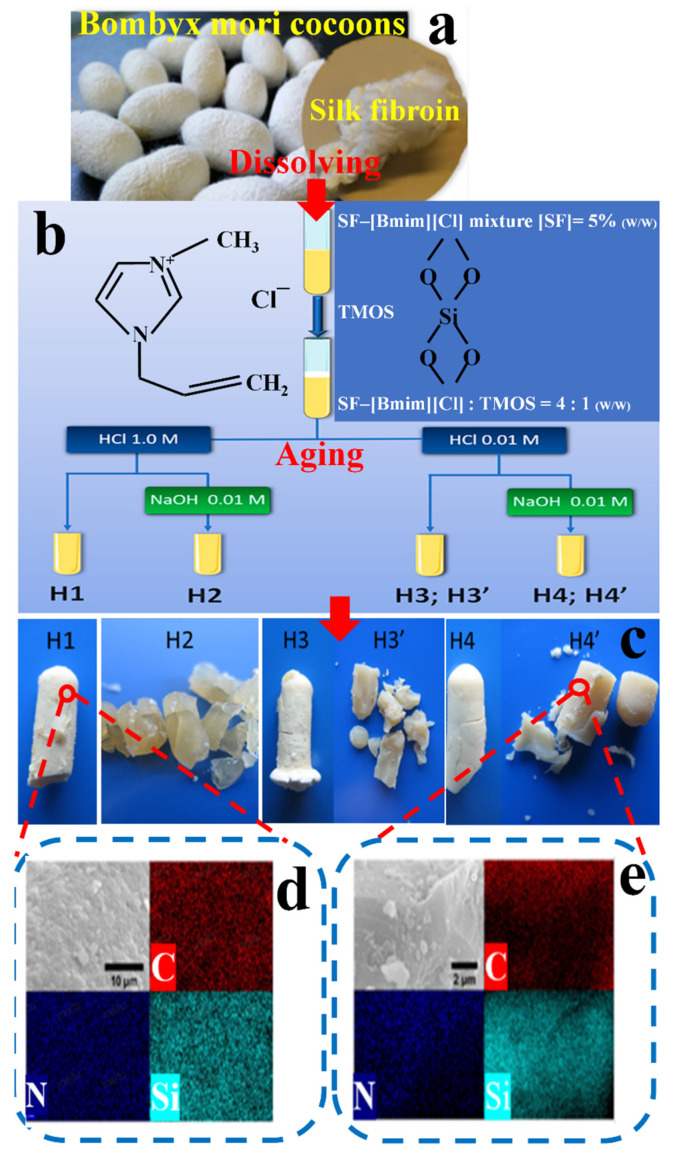
The scheme of preparation process of (**a**) SF (**b**) dissolved in [BMIM]Cl, blended with tetramethoxysilane (TMOS), and aged under different acidbase ratios. (**c**) Their appearance photos and (**d**,**e**) SEM images of H1 (10 μm) and H4′ (2 μm) at different scales, as well as corresponding EDX maps (red, navy blue and light blue in EDX map correspond to C, N and Si atoms respectively) (reproduced with permission from [115], https://pubs.acs.org/doi/10.1021/acsomega.8b02051, 7 September 2018, open access copyright (2018) American Chemical Society).

Highly ordered mesoporous materials are excellent candidates for tissue engineering, with the ability to provide controlled and local drug delivery and with the function of improving biological activity at the implant site [125]. New mesoporous hybrid materials can be obtained by dissolving inorganic materials and SF with IL, which has a broad application prospect in bone tissue engineering. Meanwhile, it can also be used as solid-state electrochemical devices, especially the electrolyte of fuel cells [115]. Figure 8 displays the preparation scheme of SF/silica biomaterial with a high surface area synthesized by using [BMIM]Cl [115]. The degummed silk (Figure 8a) was first dissolved in IL and mixed with tetramethoxysilane (TMOS) to obtain a blend solution (Figure 8b). Then, acid (HCl, 1.0 or 0.01 M) and base (NaOH, 0.01 M) as catalysts were individually added to the SF/TMOS solution. Following cured in an oil bath at 90 °C for 2 days (H1, H2, H3 and H4) and 7 days (H3′ and H4′), respectively (Figure 8c), novel mesoporous hybrid biomaterials have been obtained. Among them, IL is both a solvent of SF and a mesoporous inducer. The prepared material is slightly transparent-opaque, with amorphous monoliths with a rough surface, which was easily transformed into powder, and its stability reached about 300 °C (Figure 8c). Its energy dispersive X-ray (EDX) maps (Figure 8d,e) showed well compatibility of two-phase materials with mesopores on the micron scale. Wang et al. [116] used a natural silk cocoon as raw material to prepare porous silk carbon (Silk C) through carbonization and KOH activation in the ILs system. Their prepared silk C has a high specific surface area (S_BET_: 2854.53 m^2^ g^−1^), a large number of holes (1.54 cm^3^ g^−1^) and uniform micropores (2.5 nm). Additionally, through non-covalent π–π interaction, porous metal-free silk carbon–IL (Silk C-IL) composite could be obtained by modifying silk C in 1-butyl-3-methylimidazole hexafluorophosphate ([BMIM]PF_6_) system. This material demonstrated a good performance in constructing an electrochemical sensor for the determination of dopamine. Rath et al. [117] made a composite film used as the electrode of a flexible supercapacitor by dissolving silk fiber into 1-ethyl-3-methylimidazolium tetrafluoroborate ([EMIM]BF_4_) and being coated on the reduced graphene oxide film of cobalt (Co) doped. 

## 4. Conclusions and Prospect

Compared with traditional methods, ILs as solvents show great advantages in improving the properties of SF and its composites. These materials show good applications prospect in several fields such as tissue engineering, electrochemistry and biomedicine. Understanding the method, mechanism and main influencing factors of ILs dissolving SF will help us to innovate many new preparation methods of biomaterials with regulation properties and structures and expand more high-performance natural materials for different purposes. In this review, we introduced the SF structure and its traditional dissolving methods and expounded upon various ILs for dissolving SF and its composites with natural biological protein, inorganic matter, synthetic polymer, carbon nanotube and graphene oxide in the IL solution system as well as their applications. The main factors influencing the features of these materials were also discussed.

However, the price of ILs is much higher compared to that of traditional solvents. Additionally, for most ILs, the SF complete dissolution usually requires being stirred continuously for several hours and heated to 90–100 °C, which may lead to damage to the protein integrity and decrease the mechanical properties of biomaterials. Moreover, the high viscosity of most ILs at room temperature also limits their applications recently. Although more and more scholars have investigated the ILs dissolving SF for biomedicine and tissue engineering applications, there are many problems to be solved [117]. Among these, it is necessary to further optimize the preparation process for ILs to reduce its cost. Moreover, according to the structure of SF and the role of cation and anion in ILs, more ILs with relatively lower melting point, low viscosity and stronger hydrogen bond acceptance ability need to be designed, and the appropriate dissolution temperature and time need to be studied, which are associated with the degradation of SF, so as to create favorable conditions for the preparation of regenerated SF materials with excellent properties [126,127]. Thirdly, the morphology, the properties and the mechanism of SF and SF-based composites dissolution in various ILs need further study. In addition, it is essential to explore how the evolution of biocompatible ionic liquids would affect the polymer/protein processability. Furthermore, the use of mixtures of ionic liquids and other solvents should be on the dissolution of silk fibroin. We envisage that with many new ILs being developed, optimizing and upgrading more novel, various shapes and polyfunctional SF-based composites could happen in the near future, which will be not only used in medical biomedical sciences but also broadly useful in environmental and energy engineering, as well as various sustainable fields.

## Figures and Tables

**Table 1:** Applications of SF-based composites fabricated using various ILs *.

Components	Solvent	Shape	Applications	References
SF	[EMIM]Ac *^Anp^*	Hydrogels	Biomedical materials	[49]
	[EMIM]AC *^Bom^*	Gels	Conductive gel, biomedical materials	[49,101]
	[BMIM]AC *^Bom^*	Films	Tissue engineering materials	[63,82,83]
	[AMIM]Cl *^Bom^*	Regenerated silk Fiber	Tissue engineering materials	[81]
	[BMIM]Br *^Bom^*	Films	Tissue engineering materials	[83]
	[BMIM]Cl *^Bom^*	Films	Artificial skin coating	[82,97,98]
	[BMIM]Cl *^Ana^* [BMIM]Ac *^Ana^*	Films	Tissue engineering materials	[99]
	[BMIM]Ac *^Eri^*	Sponge	Cartilage-related biomedical materials	[102]
SF-cellulose	[EMIM]Cl	Films	Electrode materials	[80]
	[BMIM]Br [BMIM]MeSO_3_	Films	Biomedical materials	[90]
	[AMIM]Cl	Films	Tissue engineering materials	[90,103,104]
	[AMIM]Cl	Films	Biomedical materials	[103]
	[BMIM]Cl	Films	Biomedical materials	[105,106]
	[EMIM]AC	Nanoparticles, Films	Coagulation materials, Electrode materials, Tissue engineering materials	[80] [87] [107,108]
	[BMIM]Cl	Fiber	biomedicine and tissue materials	[109]
SF-carbon nanotubes	[EMIM]Cl	Films	Tissue engineering materials	[40]
SF-naringin	[EMIM]AC	Nanoparticles	Drug delivery materials	[85]
SF-chitin	[AMIM]Cl	Films	Biomedical materials	[90]
SF-KCl	[EMIM]AC	Gels	Flexible ionic conductive hydrogel	[96]
SF-Glycerol & Dimethyl sulfoxide	[BMIM]PF_6_	Films	Electrolyte film, Energy materials	[100]
SF-sucrose acetate isobutyrate	[BMIM][Ac]	Scaffolds	Tissue engineering materials	[110]
SF-cholinium gallate	Bio-ILs	Sponge	Biomedical materials	[111]
SF-chitosan	[AMIM]Cl [BMIM]Ac	Films Hydrogels	Tissue engineering materials Biomedical materials	[90,109]
SF-polylactic acid	[BMIM]Cl	Films	Drug delivery materials	[112]
SF-polyurethane	[BMIM]Cl	Films	Coagulation material	[113]
SF- polyvinyl alcohol	[AMIM]Cl	Films	Tissue engineering materials	[114]
SF-silica	[BMIM]Cl	3D scaffolds	Bone tissue materials	[82,115]
SF-carbon	[BMIM]PF_6_	3D scaffolds	Electrochemical sensor	[116]
SF-graphene oxide	[EMIM]BF_4_	Films	Electrode material	[117]

* Abbreviations: 1-allyl-3-methylimidazole chloride, [AMIM]Cl; 1-butyl-3-methylimidazole chloride, [BMIM]Cl; 1-ethyl-3-methylimidazole chloride, [EMIM]Cl; 1-ethyl-3-methylimidazole acetate, [EMIM]AC; 1-butyl-3-methylimidazole bromide, [BMIM]Br; 1-ethyl-3-methylimidazole tetrafluoroborate, [EMIM]BF_4_; 1-allyl-3-methylimidazole chloride, [AMIM]Cl; 1-butyl-3-methylimidazole hexafluorophosphate, [BMIM]PF_6_; 1-butyl-3-methylimidazole acetate, [BMIM]AC; 1-butyl-3-methylimidazole bromide, [BMIM]Br; 1-butyl-3-methylimidazole methanesulfonate, [BMIM] MeSO_3_; choline-gallate, Bio-ILs. *The superscript ‘Bom’, ‘Ana’, ‘Anp’ and ‘Eri’* represents that SF from *Bombyx mor, Antheraea assamensis, Antheraea pernyi* *and Eri silkworm cocoons can be* dissolved in this IL, *respectively.*

## Data Availability

Not applicable.
